# Preclinical Evaluation of Carfilzomib for Infant *KMT2A*-Rearranged Acute Lymphoblastic Leukemia

**DOI:** 10.3389/fonc.2021.631594

**Published:** 2021-04-15

**Authors:** Laurence C. Cheung, Rebecca de Kraa, Joyce Oommen, Grace-Alyssa Chua, Sajla Singh, Anastasia M. Hughes, Emanuela Ferrari, Jette Ford, Sung K. Chiu, Ronald W. Stam, Ursula R. Kees, Sébastien Malinge, Rishi S. Kotecha

**Affiliations:** ^1^ Division of Children’s Leukaemia and Cancer Research, Telethon Kids Cancer Centre, Telethon Kids Institute, University of Western Australia, Perth, WA, Australia; ^2^ Curtin Medical School, Curtin University, Perth, WA, Australia; ^3^ PathWest Laboratory Medicine WA, Perth, WA, Australia; ^4^ Princess Máxima Center for Pediatric Oncology, Utrecht, Netherlands; ^5^ Department of Clinical Haematology, Oncology, Blood and Marrow Transplantation, Perth Children’s Hospital, Perth, WA, Australia

**Keywords:** infant, acute lymphoblastic leukemia, *KMT2A*, *MLL*, carfilzomib, PER cell lines

## Abstract

**Background:**

Infants with *KMT2A*-rearranged B-cell precursor acute lymphoblastic leukemia (ALL) have poor outcomes. There is an urgent need to identify novel agents to improve survival. Proteasome inhibition has emerged as a promising therapeutic strategy for several hematological malignancies. The aim of this study was to determine the preclinical efficacy of the selective proteasome inhibitor carfilzomib, for infants with *KMT2A*-rearranged ALL.

**Methods:**

Eight infant ALL cell lines were extensively characterized for immunophenotypic and cytogenetic features. *In vitro* cytotoxicity to carfilzomib was assessed using a modified Alamar Blue assay with cells in logarithmic growth. The Bliss Independence model was applied to determine synergy between carfilzomib and the nine conventional chemotherapeutic agents used to treat infants with ALL. Established xenograft models were used to identify the maximal tolerated dose of carfilzomib and determine *in vivo* efficacy.

**Results:**

Carfilzomib demonstrated low IC_50_ concentrations within the nanomolar range (6.0–15.8 nm) across the panel of cell lines. Combination drug testing indicated *in vitro* synergy between carfilzomib and several conventional chemotherapeutic agents including vincristine, daunorubicin, dexamethasone, L-asparaginase, and 4-hydroperoxycyclophosphamide. *In vivo* assessment did not lead to a survival advantage for either carfilzomib monotherapy, when used to treat both low or high disease burden, or for carfilzomib in combination with multi-agent induction chemotherapy comprising of vincristine, dexamethasone, and L-asparaginase.

**Conclusions:**

Our study highlights that *in vitro* efficacy does not necessarily translate to benefit *in vivo* and emphasizes the importance of *in vivo* validation prior to suggesting an agent for clinical use. Whilst proteasome inhibitors have an important role to play in several hematological malignancies, our findings guard against prioritization of carfilzomib for treatment of *KMT2A*-rearranged infant ALL in the clinical setting.

## Introduction

The last 70 years have seen significant progress in the treatment of children with acute lymphoblastic leukemia (ALL). Initially considered an incurable disease, 5-year overall survival rates are now over 90%. This has largely been achieved through successive randomized clinical trials facilitated by international co-operative study groups, with risk stratification of patients and treatment adaptation based on presenting features, blast genetics, and early response to therapy (1). However, there remain several subgroups who continue to have significantly inferior outcomes. Infants, defined as less than 1 year of age at diagnosis, remain notoriously difficult to treat. Infants with B-cell precursor ALL harbor a *KMT2A*-rearrangement in up to 80% of cases, which is an aggressive driver mutation associated with chemo-resistance, high rates of relapse, and a 5-year event-free survival (EFS) of less than 40% (2–4). In addition, infants are vulnerable to the toxic effects of conventional chemotherapeutic agents, necessitating dose-limitations during therapy (2). As such, there is an urgent need to identify novel agents to improve outcome of infants with *KMT2A*-rearranged ALL without further increasing toxicity. In this study, we aimed to investigate the potential of the selective proteasome inhibitor carfilzomib, for the treatment of infants with *KMT2A*-rearranged ALL.

## Materials and Methods

### Cell Culture and Differentiation

A panel of eight human cell lines were established as previously described from bone marrow or peripheral blood leukocytes of infants with *KMT2A*-rearranged ALL (5). Following informed consent from a parent/guardian, primary patient specimens were sourced from infants enrolled on legacy Children’s Oncology Group (COG) trials in Perth, Western Australia. Briefly, cells obtained at the time of diagnosis or relapse were collected in preservative-free heparin and separated on Ficoll-Hypaque (Pharmacia Fine Chemicals AB, Uppsala, Sweden) at 841 × g for 25 min. Washed cells at a concentration of 1 × 10^6^/ml were placed in round bottom 96-well culture plates (Falcon, Corning, NY, USA) seeding 100 ul per well. The cells were cultured in RPMI 1640 medium (Life Technologies, CA, USA) supplemented with L-glutamine (2 mM final concentration) (Sigma-Aldrich, MO, USA), 2-mercaptoethanol (50 µM final concentration) (Sigma-Aldrich, MO, USA), sodium pyruvate (1 mM) (MP Biomedicals, CA, USA), 1% non-essential amino acids (MP Biomedicals, CA, USA), and 20% heat-inactivated fetal calf serum (FCS) (Flow Laboratories, Irvine, Scotland). The cells were cultured in a humidified 37°C incubator containing 5% CO_2_ in air. Fifty percent of the medium was replaced by fresh medium every third day. Cultures were passaged provided they had reached cell concentrations of 2 × 10^6^/ml. Cells were maintained in RPMI 1640 containing 20 or 10% FCS, 1% non-essential amino acid mix (100×), and 1% 100 mM sodium pyruvate solution. Cell viability was assessed through live cell counting by exclusion of 0.2% Trypan blue (Sigma-Aldrich, MO, USA). For all experiments, cells were seeded at pre-determined densities and incubated at 37°C and 5% CO_2_.

The cell lines are presently growing in 24-well cell culture plates (Nunclon, Thermo Fisher Scientific, MA, USA) and are passaged twice a week. The cell lines are repeatedly shown to be free of mycoplasma using the MycoAlert™ PLUS Mycoplasma Detection Kit (Lonza, Basel, Switzerland). DNA Fingerprinting was performed on the primary patient samples and on an annual basis for cell line authentication by the Australian Genome Research Facility (Melbourne, Australia), using the GenePrint 10 kit (Promega, WI, USA).

### Cell Line Characterization

The AIEOP-BFM consensus antibody panel for pediatric ALL was used to assess the immunophenotype of the cell lines by flow cytometry (6). The cells were stained with manufacturer recommended quantities of fluorochrome-conjugated antigen-specific monoclonal antibodies in 5 ml polystyrene round bottom tubes (Falcon, Corning, NY, USA). The cells were incubated with the antibodies for 20 min at room temperature shielded from light. Phosphate-buffered saline (PBS) containing 2% FCS was added to the tubes and then centrifuged at 473 × g for 5 min. The supernatant was discarded and the cells were resuspended in 300 µl of PBS-2% FCS and used for flow cytometry analysis. For intracytoplasmic antigens, cells were fixed and permeabilized using the Fixation/Permeabilization Solution Kit (BD Cytofix/Cytoperm, BD Biosciences, CA, USA) as per manufacturer instructions. A comprehensive antibody panel with 6–10 color direct immunofluorescent labeling was used, according to manufacturer recommended volumes, for immunophenotyping with a BD LSRFortessa™ X-20 flow cytometer (BD Biosciences, CA, USA) (**Supplementary Table 1**). Cell populations of interest were gated on the basis of light scattering properties. Where appropriate, unstained cells, isotype controls, or cells known to lack certain cell surface markers were used as appropriate negative controls to set quadrant gates. Conversely, cells known to express particular markers (e.g. Jurkat E6-1) were used as positive controls where required. The percentage of antigen-positive cells was decided using a combination of quadrant statistics and ranged histogram gates.

The cell lines were cultured using unsynchronized cell cultures and G-band conventional analysis was performed on metaphase chromosomes according to standard methods. Fluorescent *in situ* hybridization (FISH) testing using a commercially available KMT2A (MLL) break apart probe to screen interphase cells was carried out according to the manufacturer’s instructions (MetaSystems, Altlussheim, Germany). Directed metaphase and sequential FISH studies using whole chromosome paints and other FISH probes were performed to further characterize complex rearrangements. Chromosomal abnormalities were defined and reported according to the International System for Human Cytogenetic Nomenclature (7).

### Assessment of *In Vitro* Sensitivity and Synergy

*In vitro* cytotoxicity assays were performed using a modified Alamar Blue (resazurin reduction) assay with cells in logarithmic growth. All *in vitro* assays were performed in triplicate in 384-well clear tissue-culture treated plates (Corning, NY, USA). Cells were seeded at pre-determined optimal seeding densities (**Supplementary Table 2**) in 50 µl media using an automated Multidrop Combi Reagent Dispenser (Thermo Fisher Scientific, MA, USA). Cells were treated with drugs dispensed by the automated Tecan D300e Digital Dispenser (Tecan Group Ltd., Männedorf, Switzerland) at concentration ranges listed in **Supplementary Table 2** at fixed ratios. Drugs were dissolved in dimethyl sulfoxide (DMSO) or water for those with poor DMSO solubility or stability (**Supplementary Table 3**). Final DMSO concentration in assay wells receiving drugs dissolved in DMSO was 0.5%. After 72 h drug exposure at 37°C and 5% CO_2_, Alamar Blue reagent comprising resazurin, methylene blue, potassium hexacyanoferrate (III), and potassium hexacyanoferrate (II) (Sigma-Aldrich, MO, USA) was added (10% v/v) and cell viability determined by absorbance (570 and 600 nm) 6 h after addition of Alamar Blue using a Synergy™ Mx microplate reader (Biotek, VT, USA). Raw absorbance readings were used to generate dose-response curves. Half maximum inhibitory concentrations (IC_50_) for each compound were calculated in Microsoft Excel by plotting a log dose-response curve utilizing the slope from the linear trendline to determine the IC_50_. Cell viability was reported as a percent of the untreated control cells for each cell line.

For the synergy assays, we used a 14 × 7 pairwise checker-board matrix format that assessed each of the nine conventional chemotherapy agents used to treat infants with ALL and carfilzomib, at 13 and 6 concentrations respectively, using a series of twofold dilutions. The fraction of cells affected was calculated for each drug concentration and 72 h percent viability imputed from the growth curves. Excess over Bliss as a measurement for synergy was calculated by the Bliss Independence model using the Chalice Bioinformatics Software (Horizon Discovery, Waterbeach, United Kingdom) (8, 9).

### Assessment of *In Vivo* Efficacy

Female NOD/SCID mice were purchased from the Animal Research Centre, Perth. Animals were housed under pathogen-free conditions and all studies were approved by the Animal Ethics Committee, Telethon Kids Institute, Perth (Ethics number #312). Seven-week-old mice were inoculated with 1 × 10^6^ PER-785, 1 × 10^6^ PER-826, 1 × 10^6^ MLL-5, or 2 × 10^6^ MLL-14 cells. PER-785 and PER-826 xenografts were established from our cell line panel, whereas MLL-5 and MLL-14 are well characterized patient-derived xenograft models (10–12). Carfilzomib (Selleckchem, Houston, USA) was formulated in an aqueous solution of sulfobutylether-beta-cyclodextrin (Captisol^®^, CyDex Pharmaceuticals, CA, USA) or 2-hydroxypropyl-beta-cyclodextrin (Sigma-Aldrich, MO, USA). The maximum tolerated dose (MTD) of carfilzomib was determined in the PER-785 xenograft. Drug treatment was commenced when the percentage of human CD19^+^ CD45^+^ cells reached 1% in the bone marrow, identified from extensive mapping of leukemia cell kinetics (10). Following a 12-day engraftment period, mice were randomized into seven groups of five mice. The delivery schedule was designed to mimic that of a recently completed phase 1b study of carfilzomib in combination with induction therapy for children with relapsed/refractory ALL (13). Carfilzomib was administered intravenously *via* tail vein injection on days 1, 2, 8, 9, 15, and 16 with treatment groups comprising of 1.5, 2.0, 2.5, 3.0, 3.5, or 4.0 mg/kg dosing of carfilzomib or vehicle control. Mice were sacrificed 3 weeks from the start of therapy and leukemia burden was determined by measuring the percentage of human CD19^+^ CD45^+^ cells in the bone marrow, spleen, and peripheral blood by flow cytometry with anti-human CD19-APC and CD45-PE antibodies.

All four xenograft models were used to determine the response to drug treatment by EFS and leukemia burden in lymphoid organs post-treatment. The PER-785 and PER-826 xenograft models were used to evaluate the clinical potential of carfilzomib in the setting of minimal disease. For each xenograft model, mice were randomized into two groups of eight mice, comprising of a carfilzomib treatment group and vehicle control. Carfilzomib monotherapy was administered at the established MTD, commencing on day 12 following injection of cells for the PER-785 xenograft and on day 18 for the PER-826 xenograft, corresponding to 1% human CD19^+^ CD45^+^ cells in the bone marrow. MLL-5 and MLL-14 xenografts were used to evaluate the effect of carfilzomib at high leukemic burden and in combination with conventional chemotherapy. For each xenograft model, treatment commenced on day 17 following injection of cells for MLL-5 and on day 21 for MLL-14, corresponding to 1% human CD19^+^ CD45^+^ cells in the peripheral blood. Individual mouse EFS was calculated as the time in days from treatment initiation until mice reached a humane end point with evidence of leukemia-related morbidity.

### Assessment of Proteasome Activity

For *in vitro* assessment of proteasome activity, the eight cell lines were seeded in 24-well plates and incubated with carfilzomib at their respective IC_50_ concentrations for 72 h. Untreated cell lines incubated under the same conditions were used as a control. Cells were subsequently harvested and washed and proteasome activity was measured using the Abcam Proteasome Activity Assay Kit (Abcam, Cambridge, United Kingdom) according to manufacturer’s instructions.

For *in vivo* assessment of proteasome activity, seven-week-old mice were inoculated with 1 × 10^6^ MLL-5 cells. Drug treatment was commenced when the percentage of human CD19^+^ CD45^+^ cells reached 1% in the peripheral blood. Mice were randomized into two groups of five mice, comprising of a carfilzomib treatment group and vehicle control. A 1.5 mg/kg dose of carfilzomib was administered intravenously *via* tail vein injection on days 1 and 2. Blood was collected from all mice 10 min after the second dose was administered to the carfilzomib treatment group and red cell lysis performed using BD Pharm Lyse™ lysing buffer (BD Biosciences, CA, USA). Proteasome activity was measured using the Abcam Proteasome Activity Assay Kit (Abcam, Cambridge, United Kingdom) according to manufacturer’s instructions.

### Statistical Analyses

Statistical analyses and graphics were performed using Prism 8 (GraphPad, La Jolla, CA, USA). Data were analyzed using the two-tailed unpaired Student’s t-test. Survival studies were analyzed using log-rank test. A p value <0.05 was considered statistically significant.

## Results

### Characteristics of a Panel of Infant *KMT2A*-Rearranged Cell Lines

Eight cell lines were established from six infants with *KMT2A*-rearranged ALL (**Table 1**). Four of the infants were less than 90 days old at diagnosis, with one infant diagnosed with congenital leukemia on day 9 of life. A cell line was established at both diagnosis (PER-494) and relapse (PER-485) from one infant (P272). Two cell lines were derived at diagnosis (PER-784 and PER-826) from P337 and the MLL-14 patient-derived xenograft was also established from this infant at diagnosis (14). Immunophenotyping with the AIEOP-BFM consensus antibody panel revealed B-lineage for PER-494, PER-784, PER-826, PER-785, and PER-910, corresponding with the immunophenotypic classification from the original patient (**Table 2**). The dominant lineage assignment for PER-490 was B-cell precursor, however there was co-expression of myeloid markers in keeping with the primary patient, P287, from which it was derived. Co-expression of B and myeloid markers was also seen at relapse for patient P272 and at diagnosis for patient P810. Subsequent to *in vitro* selection pressure, the corresponding cell lines, PER-485 and PER-703, exhibit a myeloid dominant immunophenotype (**Table 2**). Cytogenetic analysis identified involvement of the same *KMT2A*-translocation partner between each cell line and corresponding primary patient sample (**Table 3**). Sideline clones were identified in several cell lines, with structural and numerical changes that were undetected in patient specimens. This could be explained by less advanced techniques for routine standard of care cytogenetic analysis when the infants were originally diagnosed or may reflect clonal evolution *in vitro*. The former can be exemplified by the initial diagnostic karyotype of P337 revealing 49,XX,+X,+X,+6[5]/46,XX[16] in 1994, with the complex three-way translocation specified in **Table 3** identified in the MLL-14 xenograft and on re-analysis of the corresponding primary patient material using more advanced techniques in 2008 (14). The minor cytogenetic clone identified in the patient was detected in the corresponding cell lines, PER-784 and PER-826. DNA fingerprinting revealed identical short tandem repeat profiles between the primary patient sample and corresponding cell line, except for single markers at the CSF1PO locus for PER-784 and PER-785 and at the TH01 locus for PER-703 (**Supplementary Table 4**). The high match percentage confirmed authentication of the cell lines.

**Table 1:** Clinical characteristics of six infants with *KMT2A*-rearranged acute lymphoblastic leukemia and corresponding cell lines.

Patient ID	Sex	Age (days) at diagnosis	WBC at presentation (×10^9^/L)	CNS status at diagnosis	Upfront therapy	Relapse	Time (months) from diagnosis to relapse	Relapse therapy	Outcome	Corresponding cell line^*^
P272	Female	336	317.0	CNS1	CCG 1883	BM	3	CCG 1008	Died of Disease; OS 5 months	PER-494 PER-485 (Relapse)
P287	Female	9	111.8	CNS2	CCG 1883	BM	3	CCG 0922	Died of Disease; OS 4 months	PER-490
P337	Female	82	564.0	CNS2	CCG 1901 → HSCT	BM	16	CCG 1882 → HSCT2	Alive without disease; 26 years of age at last follow-up	PER-784 PER-826
P399	Female	66	670.0	CNS1	CCG 1953	No	_	_	Alive without disease; 24 years of age at last follow-up	PER-785
P810	Female	52	102.0	CNS2	COG P9407 → HSCT	No	_	_	Died from hepatic sinusoidal obstruction syndrome post HSCT; OS 4 months	PER-703
P899	Male	365	156.6	CNS1	COG AALL0631	No	_	_	Alive without disease; 6 years of age at last follow-up	PER-910

*All cell lines were derived at diagnosis except for PER-485 which was derived at first relapse.

BM, Bone Marrow; CCG, Children’s Cancer Group; COG, Children’s Oncology Group; CNS, Central Nervous System; OS, Overall Survival; WBC, White Blood Cell.

**Table 2:** Immunophenotype of primary patient samples and corresponding cell lines.

Antibody	Patients and Corresponding Cell Lines														
	P272	PER-494	P272 at Relapse	PER-485	P287	PER-490	P337	PER-784	PER-826	P399	PER-785	P810	PER-703	P899	PER-910
CD1a		0%		0%		0%		0%	0%		0%		<1%		0%
CD2	7%	<1%	8%	<1%	3%	<1%	<1%	<1%	<1%	<1%	<1%	4%	<1%		<1%
iCD3		<1%		<1%		<1%		<1%	<1%		<1%	<1%	<1%	<1%	<1%
CD3	8%	<1%	7%	<1%	3%	<1%	1%	<1%	<1%	<1%	1%	4%	<1%		<1%
CD4		<1%		100%		2%		<1%	<1%		28%		99%		<1%
CD5	7%	1%	6%	<1%	3%	<1%	<1%	<1%	<1%	<1%	8%	2%	2%		<1%
CD7	6%	<1%		<1%	3%	<1%	14%	1%	99%	1%	37%	13%	<1%	11%	23%
CD8		<1%		<1%		<1%		<1%	<1%		1%		98%		<1%
CD10	84%	100%	7%	4%	0%	<1%	<1%	10%	1%	<1%	<1%	3%	29%	25%	6%
CD11b		19%		65%		30%		3%	4%		14%		100%		<1%
CD11c		17%		60%		39%		1%	24%		3%		57%		<1%
CD13	0%	16%	57%	31%	7%	99%		1%	5%	10%	74%	65%	44%		1%
CD14		<1%		11%	7%	<1%		<1%	<1%		<1%		100%		<1%
CD15		<1%	11%	100%	47%	99%		<1%	<1%		29%		100%		<1%
CD19	90%	100%	69%	<1%	72%	100%	98%	98%	99%	99%	100%	95%	<1%	96%	99%
CD20		<1%		<1%		<1%		6%	<1%		<1%	< 1%	<1%	<1%	<1%
iCD22		<1%		<1%		<1%		<1%	<1%		<1%		<1%		<1%
CD22		<1%		<1%		<1%		<1%	<1%		<1%		<1%		<1%
CD24	90%	100%		100%	11%	100%	< 1%	100%	99%	48%	100%	87%	100%		94%
CD33	0%	4%	66%	99%	77%	23%	16%	20%	50%		17%	58%	100%	1%	6%
CD34	94%	<1%	67%	<1%	21%	<1%	66%	<1%	<1%	98%	<1%	2%	<1%	2%	<1%
CD38		98%		<1%		71%		97%	100%		80%		96%	99%	99%
CD45		96%		99%		1%	100%	68%	100%		100%	100%	100%		99%
CD64		12%		17%		11%		2%	9%		20%	1%	100%		<1%
CD65		<1%		86%		66%		<1%	<1%		13%		96%		<1%
iCD79a		73%		<1%		88%		79%	94%		83%	98%	<1%	99%	90%
CD117		0%		0%		0%		0%	0%		0%	<1%	0%	3%	0%
HLA-DR	86%	99%		6%	80%	100%		100%	100%		100%	42%	95%	96%	100%
iLysozyme		0%		1%		0%		0%	0%		0%		<1%		0%
iMPO		<1%		2%		<1%		<1%	<1%		0%	1%	24%	<1%	<1%
iIgM		<1%		<1%		<1%		<1%	<1%		<1%	49%	2%	97%	<1%
IgM	1%	<1%	23%	<1%		<1%	6%	<1%	<1%		2%		<1%	12%	2%
iTDT		<1%		<1%		<1%	15%	<1%	<1%	98%	1%	6%	<1%	<1%	<1%

**Table 3:** Cytogenetic analysis of primary patient samples and corresponding cell lines.

Patient (P)/Cell Line (PER)	G-Banding Karyotype and Fluorescent *In Situ* Hybridization Analysis	Date Performed
P272	Cytogenetic analysis unsuccessful due to insufficient number of metaphases	04/1992
PER-494	46,XX,der(4)(4pter->4p1?2::7p1?5->7p13::4q21->4p1?2::7p1?5->7pter),der(7)(11qter->11q23::7p13->7qter),add(8)(q24), der(11)(11pter->11q23::4q21->4qter)[20] Chromosome 11(q23) FISH studies were abnormal. A split *KTM2A* signal was detected in 95 of 100 interphase cells scored. Directed metaphase FISH testing confirmed a three-way translocation involving chromosomes 4, 11, and 7, with 5’ proximal *KMT2A* remaining on the der(11)t(4;11;7) and 3’ distal *KMT2A* translocating to 7p on the der(7)t(4;11;7), resulting in the oncogenic derivative 11 being formed (*AFF1-KMT2A*). Note: *AFF1*, formerly known as *AF4* was not demonstrated by FISH analysis. Directed metaphase FISH testing using whole chromosome 4 and 7 paints confirmed this translocation and elucidated the subsequent inversion of the der(4)t(4;11;7) involving regions of 4 and 7 material.	02/2018
P272 at Relapse	46,XX,add(4)(q12),del(7)(p14),add(8)(q24.3),der(9)inv(9)(p11q12)del(9)(p24),der(11)t(4;11)(q21;q23)[18]/46,XX[4]	07/1992
PER-485	47,X,-X,add(1)(p36.1),-4,+5,del(5)(q12q33),+6,add(7)(p13),add(8)(q24.3),der(11)t(4;11)(q21;q23),+mar[20] Directed metaphase whole chromosome 4 and 5 FISH paints confirmed the del(5q) and the presence of chromosomal 4 material on the marker. Directed metaphase *KMT2A* BAP FISH and whole chromosome 11 paint confirmed the presence of chromosomal 11 material on the add(7p) with rearrangement of the *KMT2A* gene resulting in 5’ proximal *KMT2A* remaining on the der(11)t(4;11) and 3’ distal *KMT2A* relocating to the add(7p) abnormality.	01/2020
P287	46,XX,t(4;11)(q21;q23)[15]/46,XX[5]	10/1992
PER-490	46,X,-X,+1,dic(1;21)(p11.2;q22),t(4;11)(q21;q23)[18]/46,idem,t(8;11)(q24;q14)[3] Chromosome 11(q23) FISH studies were abnormal. A split *KMT2A* signal was detected in 191 of the 200 interphase cells scored. *KMT2A* directed metaphase FISH confirmed the t(4;11) with 3’ distal *KMT2A* located on the der(4)t(4;11) and 5’ proximal *KMT2A* remaining on der(11)t(4;11). Directed metaphase whole chromosome 8 paint and *KMT2A* FISH analysis confirmed the t(8;11) abnormality with the presence of the intact *KMT2A* gene relocated onto the long (q) arm of the der(8)t(8;11). Directed metaphase whole chromosome 1 and 21 paints and *RUNX1* breakapart FISH studies confirmed the dic(1;21) and the presence of an intact *RUNX1* signal on the dic(1;21).	10/2019
P337	46,XX,der(2)(2pter->2q37::11q23->q23::13q32->qter),der(11)(11pter->q23::19p13.3->pter),der(13)(13pter->13q32::11q23->qter), der(19)(2qter->q37::19p13.3->qter)[88/100]/46,XX,t(11;13)(q23;q32)[12/100]	01/2008
PER-784	46,XX,t(11;13)(q23;q34)[16] Chromosome 11(q23) FISH studies were abnormal. A split *KMT2A* signal was detected in 182 of the 200 cells scored. Directed metaphase *KMT2A* breakapart FISH studies confirmed the t(11;13) abnormality.	12/2019
PER-826	46,XX,t(11;13)(q23;q34),?inv(18)(p11.2)[20] Directed metaphase *KMT2A* FISH and whole chromosome 11 paint confirmed the t(11;13) abnormality.	12/2019
P399	46,XX,t(4;11)(q21;q23)[20]	11/1996
PER-785	47,XX,t(4;11)(q21;q23),+19,der(19)t(1;19)(q12;p13.3)[20] Directed metaphase *KMT2A* FISH studies confirmed the t(4;11) abnormality with the 3’ distal *KMT2A* region translocated to the short arm of the derivative 4 chromosome and the 5’ proximal region remaining on the long arm of the derivative 11 chromosome.	09/2020
P810	46,XX,t(1;11)(p32;q23)[13]/46,XX[7] Chromosome 11(q23) FISH studies were abnormal. A split *KMT2A* signal was detected in 109 of the 200 cells scored.	07/2007
PER-703	79-83,XXX,-X,der(1)t(1;11)(p32;q23),t(1;11)(p32;q23)x2,-2,-4,der(5)t(1;5)(q21;q22),+7,-9,-10,-11,-11,-12,+13,-15,-16,-17,-18,-21,-21,-22[20] Directed metaphase *KMT2A* FISH studies confirmed the presence of the two t(1;11) aberrations and the additional der(1)t(1;11) abnormality. The signal patterns demonstrated a 3’ distal signal present on the short arm of the three copies of der(1)t(1;11) and a 5’ proximal signal remaining on the long arm of the two copies of der(11)t(1;11).	09/2020
P899	46,XY,t(9;11)(p21;q23)[14]/46,XY[6]Chromosome 11(q23) FISH studies were abnormal. A split *KMT2A* signal, was detected in 174 of the 200 cells scored. Directed *KMT2A* FISH analysis confirmed the t(9;11).	11/2014
PER-910	46,XY,t(9;11)(p21;q23)[20] Directed *KMT2A* FISH confirmed the t(9,11) abnormality demonstrating a 3’ distal *KMT2A* signal translocated to the short arm of the derivative 9 chromosome and the 5’ proximal signal remaining on the long arm of the derivative 11 chromosome.	09/2020

### Carfilzomib Is Cytotoxic *In Vitro* and Exhibits Synergy With Conventional Chemotherapeutic Agents Used in Induction

**Figure 1:**
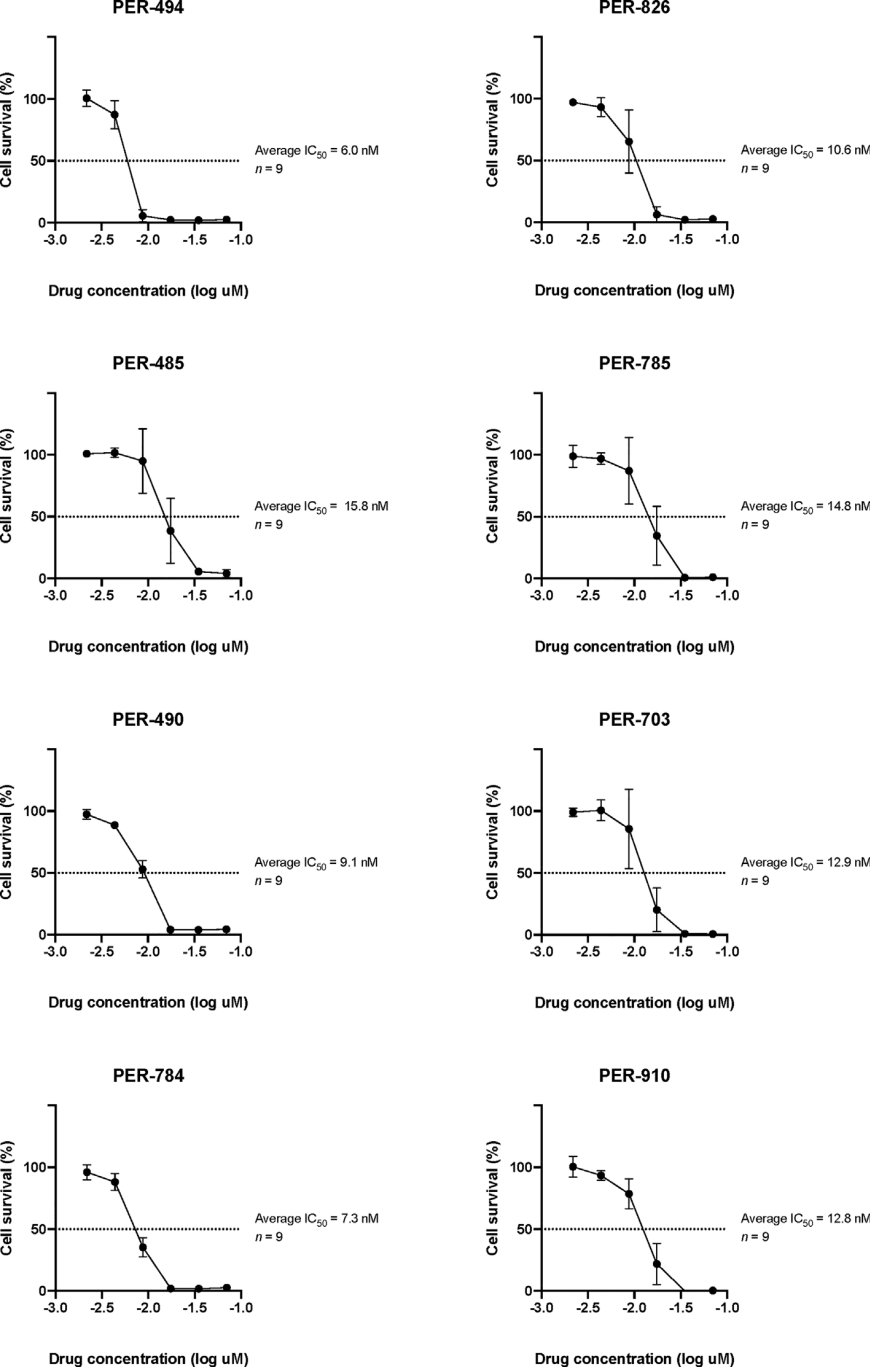
Log dose-response analyses of carfilzomib in PER cell lines derived from infants with *KMT2A*-rearranged acute lymphoblastic leukemia. The half maximum inhibitory concentrations (IC_50_) are indicated. Data points are represented as the mean ± standard deviation of n = 9 replicates.

**Figure 2:**
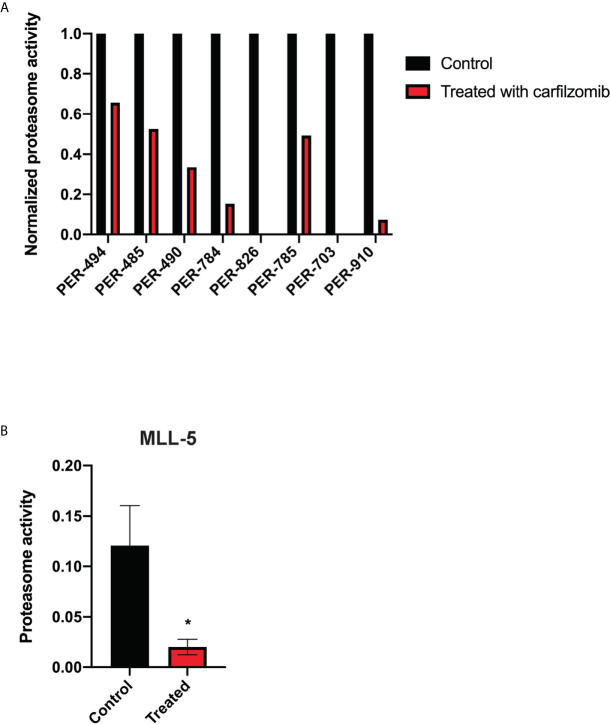
Carfilzomib inhibits proteasome activity. **(A)** Proteasome activity of PER cell lines derived from infants with *KMT2A*-rearranged acute lymphoblastic leukemia following incubation with carfilzomib at their respective half maximum inhibitory concentrations (IC_50_) for 72 h normalized to proteasome activity of untreated cell lines. **(B)** Proteasome activity in peripheral blood of MLL-5 patient-derived xenografts treated with 1.5 mg/kg of intravenous carfilzomib *via* tail vein injection on days 1 and 2 compared to untreated mice (n = 5 mice per group). Proteasome activity was measured 10 min after the injection on day 2. Mice were treated at high leukemic burden in the bone marrow corresponding to a mean peripheral blood blast percentage of 5.1% for treated mice and 4.2% for untreated mice. Error bars represent mean ± standard error of mean. *P < 0.05.

Carfilzomib demonstrated low IC_50_ concentrations within the nanomolar range across all cell lines (PER-494: 6.0 nM; PER-485: 15.8 nM; PER-490: 9.1 nM; PER-784: 7.3 nM; PER-826: 10.6 nM; PER-785: 14.8 nM; PER-703: 12.9 nM; PER-910: 12.8 nM) (**Figure 1**). Carfilzomib was shown to inhibit proteasome activity at the IC_50_ concentration in all cell lines compared to untreated control (**Figure 2A**). Combination drug testing indicated *in vitro* synergy between carfilzomib and several conventional chemotherapeutic agents including vincristine, daunorubicin, dexamethasone, L-asparaginase, and 4-hydroperoxycyclophosphamide, with six or more cell lines demonstrating positive sum of synergy scores according to Excess over Bliss (**Table 4**). Notably, the highest total synergy scores in the two cell lines with a myeloid dominant immunophenotype (PER-485 and PER-703) occurred in combination with cytarabine (**Table 4**). As a first step towards assessing the potential of such drug combinations, we examined carfilzomib as single agent *in vivo*.

**Table 4:** Total *in vitro* synergy scores between carfilzomib and conventional chemotherapy agents.

Cell Line	Vincristine	Daunorubicin	Dexamethasone	Cytarabine	Methotrexate	6-Mercaptopurine	L-Asparaginase	6-Thioguanine	4-HPC
PER-494	36.07	170.57	41.98	−24.52	−41.30	9.30	56.93	−48.40	59.60
PER-485	86.40	282.00	39.02	593.00	200.00	−211.30	320.33	36.83	386.33
PER-490	18.93	98.90	38.90	81.22	−54.10	37.77	247.67	156.10	235.00
PER-784	−192.00	−59.77	−138.00	−199.33	−302.00	−320.33	151.00	−54.16	−12.30
PER-826	162.10	71.20	196.50	68.40	−59.67	2.80	383.00	69.95	177.50
PER-785	197.67	240.00	188.00	−72.37	−52.53	−12.94	99.15	5.40	161.17
PER-703	83.00	275.00	−223.43	661.00	53.33	−311.37	−344.67	262.67	−185.07
PER-910	−6.34	269.50	240.33	23.73	−285.67	−159.50	192.00	−164.00	84.60

Colour key:									
<−100	Antagonism								
0 to −100	Mild antagonism								
0 to 100	Additive								
100 to 200	Mild synergy								
200 to 300	Synergy								
>300	Strong synergy								

### Carfilzomib Displays Toxicity *In Vivo* and Does Not Prolong Survival as a Single Agent or in Combination With Induction Chemotherapy

**Figure 3:**
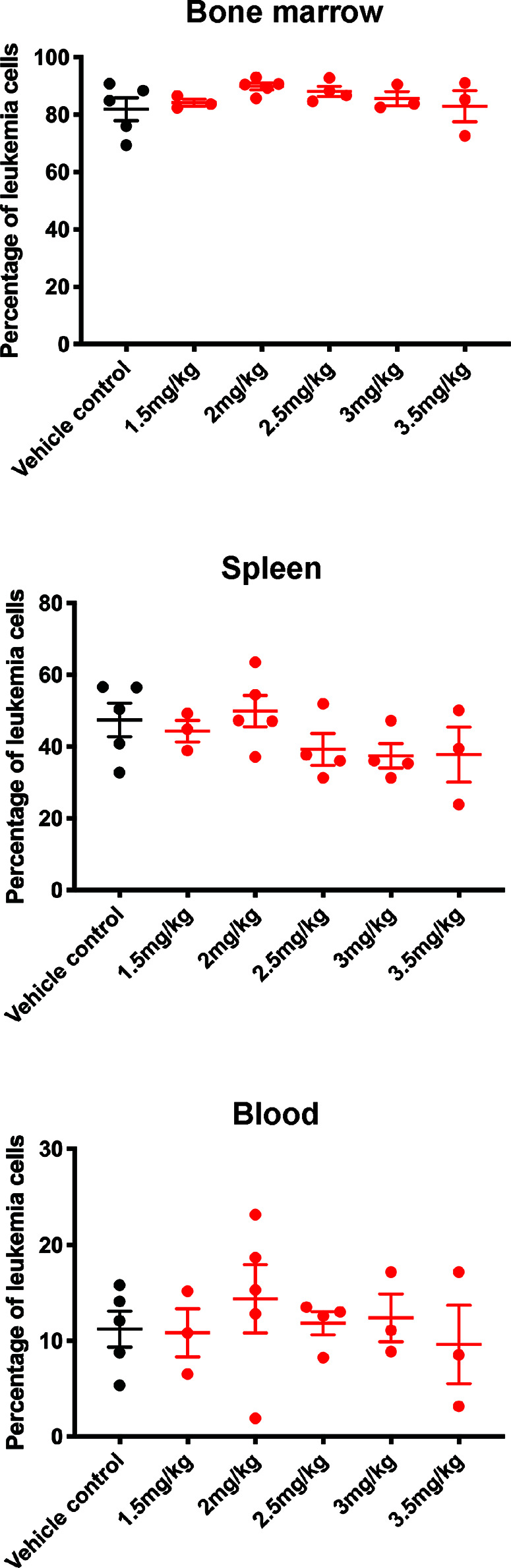
Carfilzomib monotherapy does not reduce leukemic burden in the PER-785 xenograft. Following a 12-day engraftment period, carfilzomib was administered intravenously on days 1, 2, 8, 9, 15, and 16 with treatment groups comprising of vehicle control, 1.5, 2.0, 2.5, 3.0, 3.5, or 4.0 mg/kg dosing of carfilzomib (n = 5 mice per group). Mice were sacrificed 3 weeks from the start of therapy and leukemia burden was determined by measuring the percentage of human CD19^+^ CD45^+^ cells in the bone marrow, spleen, and peripheral blood. Error bars represent mean ± standard error of mean.

**Figure 4:**
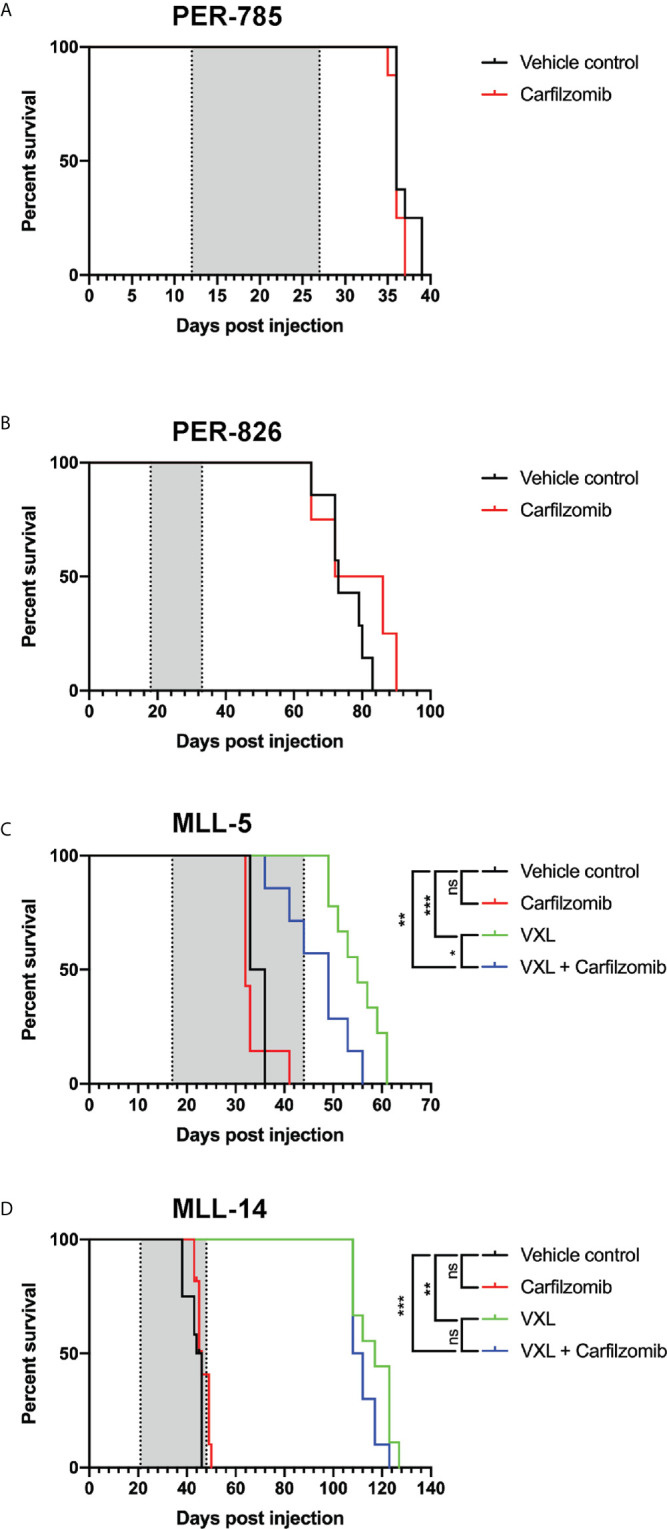
Carfilzomib does not extend survival in infant *KMT2A*-rearranged acute lymphoblastic leukemia xenografts. **(A, B)** Kaplan-Meier survival curves of mice injected with PER-785 or PER-826 leukemia cells that were treated at low leukemic burden with vehicle control or 2 mg/kg carfilzomib on days 1, 2, 8, 9, 15, and 16 (n = 8 mice per group). **(C, D)** Kaplan-Meier survival curves of mice injected with MLL-5 or MLL-14 leukemia cells that were treated at high leukemic burden with carfilzomib, VXL, carfilzomib-VXL combination therapy, or vehicle control (n = 10 mice per group). Carfilzomib was administered intravenously on days 1, 2, 8, 9, 15, and 16 at 1.5 mg/kg. VXL comprised of 0.15 mg/kg of vincristine once a week for 4 weeks, dexamethasone 5 mg/kg 5 days a week for 4 weeks, and L-asparaginase 1,000 U/kg 5 days a week for 4 weeks, administered by intraperitoneal injection. Gray shaded areas indicate treatment duration, with the first dotted line representing the start of therapy and the second dotted line the end of therapy. *P < 0.05, **P < 0.001, ***P < 0.0001.

Leukemia-bearing mice receiving 2.5 mg/kg or more of carfilzomib developed weight loss, diarrhea, shortness of breath, slower movement, and swelling at the injection site. Mice that received 4.0 mg/kg were euthanized due to excessive morbidity prior to completion of therapy, thus establishing the MTD of carfilzomib as 2.0 mg/kg. There was no difference in leukemia burden in the bone marrow, spleen, or peripheral blood between any of the doses tested or vehicle control at the defined endpoint (**Figure 3**). This finding was extended for assessment of EFS, when carfilzomib monotherapy was used to treat minimal disease burden in both the PER-785 and PER-826 xenograft models. Compared to vehicle control, no significant survival benefit was seen for carfilzomib at the MTD of 2.0 mg/kg (**Figures 4A, B**).

Results from the *in vitro* synergy screen identified positive Bliss scores in the majority of cell lines for carfilzomib and chemotherapeutic agents used during induction therapy for children with ALL (**Table 4**). Given this finding, we sought to determine whether carfilzomib could potentiate the effect of multi-agent induction therapy. This was performed using the MLL-5 and MLL-14 xenografts, as they have been extensively characterized for their response to vincristine, dexamethasone, and L-asparaginase (VXL) (12). As induction therapy is administered to children with high leukemic burden and to additionally determine whether carfilzomib could provide an *in vivo* survival benefit at high leukemic burden, treatment was commenced when the percentage of human CD19^+^ CD45^+^ cells reached 1% in the blood. For each xenograft model, mice were randomized into four groups of 10 mice, with groups comprising of carfilzomib monotherapy, VXL, carfilzomib-VXL combination therapy, or vehicle control. VXL treatment comprised of 0.15 mg/kg of vincristine (Pfizer, NY, USA) once a week for 4 weeks, dexamethasone (Mylan, PA, USA) 5 mg/kg 5 days a week for 4 weeks, and L-asparaginase (Sanofi, Paris, France) 1,000 U/kg 5 days a week for 4 weeks administered by intraperitoneal injection (15). To minimize toxicity when combined with VXL therapy, carfilzomib was administered at one dose level below the established MTD, namely 1.5 mg/kg, with *in vivo* inhibition of proteasome activity demonstrated at this dose level compared to untreated controls (**Figure 2B**). However, despite the on-target effect, no survival benefit was seen for carfilzomib, either as a single agent when compared to vehicle control, or in combination with VXL therapy compared to VXL alone in MLL-5 or MLL-14 xenografts treated with high leukemic burden (**Figures 4C, D**).

## Discussion

Infant *KMT2A*-rearranged ALL has a desperately poor outcome. The high rate of relapse combined with the dose-limiting toxicities of conventional chemotherapeutic agents has led to the search for novel agents that can be integrated into the treatment schedule. However, infant *KMT2A*-rearranged ALL is a rare disease and there is a consequent limitation to the number of trials that can be concurrently active, with prolonged periods required to meet target accrual. Currently there are four main groups conducting clinical trials for infant ALL, namely the COG, Japanese Pediatric Leukemia/Lymphoma Study Group, St Jude Children’s Research Hospital, and the INTERFANT study group. The most recently completed trial from the latter, INTERFANT-06, required 10 years to accrue 651 patients with no improvement in outcome compared to the preceding INTERFANT-99 study (3, 16). The most recent phase 3 COG study, AALL0631, added the FLT3 inhibitor lestaurtinib to post-induction chemotherapy for infants with *KMT2A*-rearranged ALL, with no improvement to overall outcome (17). Given these limitations and to ensure progress is made it is imperative that novel agents undergo rigorous and robust preclinical assessment to provide the best evidence to enable clinical trial development committees to make informed decisions regarding selection of the best agents for integration into the clinical setting.

Proteasome inhibitors have emerged as a powerful treatment strategy for multiple myeloma (18). The ubiquitin-proteasome system maintains protein homeostasis by controlling the degradation of damaged, abnormally folded, or short-lived regulatory proteins. Consequently, it has the capacity to influence key cellular functions related to progression through the cell cycle, cellular proliferation, cellular differentiation, DNA damage repair, and cell death. Elevated proteasomal activity has been demonstrated in many cancers, notably hematological neoplasms, because malignant cells are often heavily dependent on proteasomal function, particularly for the degradation of proteins that hinder proliferation. Inhibition of proteasome with proteasome inhibitors impairs turnover of multiple proteins resulting in their accumulation in the cell and disruption of multiple signaling pathways within the cell, leading to disruption of cell cycle and apoptosis.

In recent years, proteasome inhibitors have been increasingly investigated for the treatment of pediatric ALL. Bortezomib was the first-in-class reversible proteasome inhibitor to receive FDA approval and has undergone clinical investigation in the relapsed/refractory setting. Although these studies have shown that the addition of bortezomib to induction chemotherapy improves response rates, it is yet to translate into a survival advantage (19). In particular, encouraging response rates have been seen in children with relapsed T-ALL (20), resulting in development of the COG phase 3 AALL1231 study, which randomized the addition of bortezomib to standard induction therapy in patients with newly diagnosed T-ALL (NCT02112916). However, this trial was prematurely closed due to the need to integrate findings from the predecessor COG AALL0434 study which identified benefit of nelarabine for T-ALL (21). The tolerability of incorporating bortezomib and vorinostat into an ALL chemotherapy backbone for newly diagnosed infants with ALL is currently being investigated by St Jude Children’s Research Hospital in a single arm phase 2 study (NCT02553460). The results of this study will be eagerly anticipated as the addition of two novel agents, bortezomib and vorinostat, to an ALL chemotherapy backbone represents a bold strategy that is required to improve the outcome for infants with ALL. However, elucidating the magnitude of any effect specifically attributable to bortezomib may ultimately prove difficult due to concurrent administration with vorinostat and conventional chemotherapeutic agents. The COG phase 3 AAML1031 trial identified that the addition of bortezomib to standard chemotherapy increased toxicity but did not improve survival in children with acute myeloid leukemia (22).

Carfilzomib is a second-generation irreversible proteasome inhibitor and was the second agent in class to receive FDA approval for the treatment of multiple myeloma. A phase 1b study of carfilzomib in combination with induction therapy for children with relapsed/refractory ALL has recently completed accrual, with tolerability identified from preliminary findings (13). However, infants were excluded from this study precluding the ability to draw any conclusions regarding clinical use of carfilzomib in this population. Our study was able to demonstrate *in vitro* cytotoxicity of the selective proteasome inhibitor carfilzomib, against infant *KMT2A-*rearranged cell lines. Carfilzomib exhibited *in vitro* synergy with several chemotherapeutic agents conventionally used to treat infant ALL, including those administered during induction therapy. Similar to our findings, previous studies have demonstrated effective cytotoxicity of carfilzomib in commercially available pediatric leukemia cell lines, which included one commercially available infant ALL cell line (KOPN8), and in primary pediatric leukemia cells (23, 24). However, despite showing an on-target effect, our findings did not translate to an *in vivo* benefit, with higher carfilzomib doses leading to excessive toxicity and an inability to demonstrate a survival advantage for carfilzomib alone or in combination with induction-style chemotherapy in *KMT2A*-rearranged infant ALL patient-derived xenograft models. However, a limitation of the study was that proteasome activity was measured in the peripheral blood and thus the inhibitory effect of carfilzomib was representative of an effect on normal cells as well as blast cells. Proteasome activity was not measured in the bone marrow, raising the potential that carfilzomib did not reach blast cells in the bone marrow or sustain relevant therapeutic concentrations, which could also account for the lack of *in vivo* efficacy.

In conclusion, our study highlights that *in vitro* efficacy does not necessarily translate into benefit *in vivo* and emphasizes the importance of *in vivo* validation prior to suggesting an agent for clinical use. Whilst proteasome inhibitors have an important role to play in several hematological malignancies, our findings guard against prioritization of carfilzomib for treatment of *KMT2A*-rearranged infant ALL in the clinical setting. Current clinical trial development for *KMT2A*-rearranged infant ALL will therefore focus on integration of novel agents which have strong preclinical data, such as venetoclax (25) and menin inhibitors (26), and immunotherapeutic approaches with promising preliminary clinical findings, such as blinatumomab (27) and CAR T-cell therapy (28).

## Data Availability Statement

The original contributions presented in the study are included in the article/**Supplementary Material**. Further inquiries can be directed to the corresponding author.

## Ethics Statement

The animal study was reviewed and approved by Telethon Kids Institute Animal Ethics Committee.

## Author Contributions

LC, UK, and RSK conceived and designed the study. RSK provided study materials and patient information. RdK performed and interpreted the cytogenetic analysis. JF developed the cell lines. JO, AH, and EF performed *in vitro* experiments. G-AC and SS performed *in vivo* experiments. RS, SC, SM, and UK provided technical insight and analyzed results. LC and RK interpreted data and supervised the project. JO and RSK wrote the manuscript. All authors contributed to the article and approved the submitted version.

## Funding

RSK is supported by a Fellowship from the National Health and Medical Research Council of Australia (NHMRC APP1142627). SM is supported by a Fellowship from the Cancer Council Western Australia. This study was supported by project grants from the Children’s Leukaemia and Cancer Research Foundation and The Kids’ Cancer Project.

## Conflict of Interest

The authors declare that the research was conducted in the absence of any commercial or financial relationships that could be construed as a potential conflict of interest.

## References

[1] Hunger SP Mullighan CG. Acute lymphoblastic leukemia in children. N Engl J Med (2015) 373(16):1541–52. 10.1056/NEJMra1400972 26465987

[2] Kotecha RS Gottardo NG Kees UR Cole CH. The evolution of clinical trials for infant acute lymphoblastic leukemia. Blood Cancer J (2014) 4(4):e200. 10.1038/bcj.2014.17 24727996 PMC4003413

[3] Pieters R De Lorenzo P Ancliffe P Aversa LA Brethon B Biondi A. Outcome of infants younger than 1 year with acute lymphoblastic leukemia treated with the Interfant-06 protocol: results from an international phase III randomized study. J Clin Oncol (2019) 37(25):2246–56. 10.1200/JCO.19.00261 31283407

[4] Winters AC Bernt KM. MLL-rearranged leukemias - an update on science and clinical approaches. Front Pediatr (2017) 5:4. 10.3389/fped.2017.00004 28232907 PMC5299633

[5] Kees UR Ford J Price PJ Meyer BF Herrmann RP. PER-117: a new human ALL cell line with an immature thymic phenotype. Leuk Res (1987) 11(5):489–98. 10.1016/0145-2126(87)90082-8 3472019

[6] Dworzak MN Buldini B Gaipa G Ratei R Hrusak O Luria D. AIEOP-BFM consensus guidelines 2016 for flow cytometric immunophenotyping of pediatric acute lymphoblastic leukemia. Cytometry B Clin Cytom (2018) 94(1):82–93. 10.1002/cyto.b.21518 28187514

[7] McGowan-Jordan J Simons A Schmid M. ISCN 2016: an international system for human cytogenetic nomenclature. Basel: Karger (2016). 10.1159/isbn.978-3-318-06861-0

[8] Bliss CI. The toxicity of poisons applied jointly. Ann Appl Biol (1939) 26(3):585–615. 10.1111/j.1744-7348.1939.tb06990.x

[9] Greco WR Bravo G Parsons JC. The search for synergy: a critical review from a response surface perspective. Pharmacol Rev (1995) 47(2):331–85. 7568331

[10] Cheung LC Cruickshank MN Hughes AM Singh S Chua GA Ford J. Romidepsin enhances the efficacy of cytarabine in vivo, revealing histone deacetylase inhibition as a promising therapeutic strategy for KMT2A-rearranged infant acute lymphoblastic leukemia. Haematologica (2019) 104(7):e300–3. 10.3324/haematol.2018.192906 PMC6601077 30679330

[11] Cruickshank MN Ford J Cheung LC Heng J Singh S Wells J. Systematic chemical and molecular profiling of MLL-rearranged infant acute lymphoblastic leukemia reveals efficacy of romidepsin. Leukemia (2017) 31(1):40–50. 10.1038/leu.2016.165 27443263 PMC5220136

[12] Richmond J Carol H Evans K High L Mendomo A Robbins A. Effective targeting of the P53-MDM2 axis in preclinical models of infant MLL-rearranged acute lymphoblastic leukemia. Clin Cancer Res (2015) 21(6):1395–405. 10.1158/1078-0432.CCR-14-2300 PMC4359964 25573381

[13] Burke MJ Ziegler DS Sirvent FJB Attarbaschi A Gore L Locatelli F. Phase 1b study of carfilzomib in combination with induction chemotherapy in children with relapsed or refractory acute lymphoblastic leukemia (ALL). Blood (2019) 134(Supplement_1):3873. 10.1182/blood-2019-127350

[14] Henderson MJ Choi S Beesley AH Baker DL Wright D Papa RA. A xenograft model of infant leukaemia reveals a complex MLL translocation. Br J Haematol (2008) 140(6):716–9. 10.1111/j.1365-2141.2007.06966.x 18218047

[15] Szymanska B Wilczynska-Kalak U Kang MH Liem NL Carol H Boehm I. Pharmacokinetic modeling of an induction regimen for in vivo combined testing of novel drugs against pediatric acute lymphoblastic leukemia xenografts. PloS One (2012) 7(3):e33894. 10.1371/journal.pone.0033894 22479469 PMC3315513

[16] Stutterheim J van der Sluis IM de Lorenzo P Alten J Ancliffe P Attarbaschi A. Clinical implications of minimal residual disease detection in infants with KMT2A-rearranged acute lymphoblastic leukemia treated on the Interfant-06 protocol. J Clin Oncol (2021) 39(6):652–62. 10.1200/JCO.20.02333 PMC8196086 33405950

[17] Brown P Kairalla J Wang C Dreyer Z Salzer W Sorenson M. Addition of FLT3 inhibitor lestaurtinib to post-induction chemotherapy does not improve outcomes in MLL-rearranged infant acute lymphoblastic leukemia (ALL): AALL0631, a Children’s Oncology Group study. Pediatr Blood Cancer (2016) 63(S3):S7. 10.1002/pbc.26233 27077670 PMC7167840

[18] Ito S. Proteasome inhibitors for the treatment of multiple myeloma. Cancers (Basel) (2020) 12(2):265. 10.3390/cancers12020265 PMC7072336 31979059

[19] Messinger YH Bostrom BC. Bortezomib-based four-drug induction does induce a response in advanced relapsed ALL but cure remains elusive. Pediatr Blood Cancer (2020) 67(3):e28115. 10.1002/pbc.28115 31820566

[20] Horton TM Whitlock JA Lu X O’Brien MM Borowitz MJ Devidas M. Bortezomib reinduction chemotherapy in high-risk ALL in first relapse: a report from the Children’s Oncology Group. Br J Haematol (2019) 186(2):274–85. 10.1111/bjh.15919 PMC6606340 30957229

[21] Dunsmore KP Winter SS Devidas M Wood BL Esiashvili N Chen Z. Children’s Oncology Group AALL0434: a phase III randomized clinical trial testing nelarabine in newly diagnosed T-Cell acute lymphoblastic leukemia. J Clin Oncol (2020) 38(28):3282–93. 10.1200/JCO.20.00256 PMC7526719 32813610

[22] Aplenc R Meshinchi S Sung L Alonzo T Choi J Fisher B. Bortezomib with standard chemotherapy for children with acute myeloid leukemia does not improve treatment outcomes: a report from the Children’s Oncology Group. Haematologica (2020) 105(7):1879–86. 10.3324/haematol.2019.220962 PMC7327649 32029509

[23] Swift L Jayanthan A Ruan Y Anderson R Boklan J Trippett T. Targeting the proteasome in refractory pediatric leukemia cells: characterization of effective cytotoxicity of carfilzomib. Target Oncol (2018) 13(6):779–93. 10.1007/s11523-018-0603-0 30446871

[24] Niewerth D Franke NE Jansen G Assaraf YG van Meerloo J Kirk CJ. Higher ratio immune versus constitutive proteasome level as novel indicator of sensitivity of pediatric acute leukemia cells to proteasome inhibitors. Haematologica (2013) 98(12):1896–904. 10.3324/haematol.2013.092411 PMC3856965 24056819

[25] Khaw SL Suryani S Evans K Richmond J Robbins A Kurmasheva RT. Venetoclax responses of pediatric ALL xenografts reveal sensitivity of MLL-rearranged leukemia. Blood (2016) 128(10):1382–95. 10.1182/blood-2016-03-707414 PMC5016707 27343252

[26] Krivtsov AV Evans K Gadrey JY Eschle BK Hatton C Uckelmann HJ. A menin-MLL inhibitor induces specific chromatin changes and eradicates disease in models of MLL-rearranged leukemia. Cancer Cell (2019) 36(6):660–73.e11. 10.1016/j.ccell.2019.11.001 31821784 PMC7227117

[27] Clesham K Rao V Bartram J Ancliff P Ghorashian S O’Connor D. Blinatumomab for infant acute lymphoblastic leukemia. Blood (2020) 135(17):1501–4. 10.1182/blood.2019004008 32043146

[28] Annesley C Summers C Pulsipher MA Wayne AS Rivers J Lamble AJ. Clinical experience of CAR T cell immunotherapy for relapsed and refractory infant ALL demonstrates feasibility and favorable responses. Blood (2019) 134(Supplement_1):3869. 10.1182/blood-2019-131447

