# Supplementary material for: Preclinical Evaluation of Carfilzomib for Infant KMT2A-Rearranged Acute Lymphoblastic Leukemia
Source: Front Oncol. 2021 Apr 15;11:631594. doi: 10.3389/fonc.2021.631594 (PMC8082024; doi:10.3389/fonc.2021.631594)
Supplement: Supplementary file 4 [file Table_4.docx]

**Supplementary Table 4.** Summary of Short Tandem Repeat Profiles of Infants with KMT2A-rearranged Acute Lymphoblastic Leukemia and Corresponding Cell Lines

| Loci | P272 | PER-494 | PER-485 | P287 | PER-490 | P337 | PER-784 | PER-826 | P399 | PER-785 | P810 | PER-703 | P899 | PER-910 |
| --- | --- | --- | --- | --- | --- | --- | --- | --- | --- | --- | --- | --- | --- | --- |
| AMEL | X,X | X,X | X,X | X,X | X,X | X,X | X,X | X,X | X,X | X,X | X,X | X,X | X,Y | X,Y |
| CSF1PO | 11,11 | 11,11 | 11,11 | 11,11 | 11,11 | 10,12 | 10,12,13 | 10,12 | 11,11 | 11,12 | 10,12 | 10,12 | 10,12 | 10,12 |
| D13S317 | 8,12 | 8,12 | 8,12 | 11,12 | 11,12 | 11,12 | 11,12 | 11,12 | 11,12 | 11,12 | 12,13 | 12,13 | 9,13 | 9,13 |
| D16S539 | 11,11 | 11,11 | 11,11 | 11,12 | 11,12 | 11,13 | 11,13 | 11,13 | 9,9 | 9,9 | 9,11 | 9,11 | 12,14 | 12,14 |
| D21S11 | 30.2,31.2 | 30.2,31.2 | 30.2,31.2 | 31,33.2 | 31,33.2 | 27,30 | 27,30 | 27,30 | 30,30.2 | 30,30.2 | 30,31.2 | 30,31.2 | 28,30 | 28,30 |
| D5S818 | 12,12 | 12,12 | 12,12 | 11,12 | 11,12 | 11,12 | 11,12 | 11,12 | 11,12 | 11,12 | 12,12 | 12,12 | 12,13 | 12,13 |
| D7S820 | 10,11 | 10,11 | 10,11 | 10,11 | 10,11 | 11,13 | 11,13 | 11,13 | 11,13 | 11,13 | 10,11 | 10,11 | 10,10 | 10,10 |
| TH01 | 6,9 | 6,9 | 6,9 | 6,9.3 | 6,9.3 | 9.3,9.3 | 9.3,9.3 | 9.3,9.3 | 6,7 | 6,7 | 7,9.3 | 9.3,9.3 | 7,9 | 7,9 |
| TPOX | 9,9 | 9,9 | 9,9 | 8,8 | 8,8 | 8,10 | 8,10 | 8,10 | 8,8 | 8,8 | 8,9 | 8,9 | 8,11 | 8,11 |
| vWA | 15,16 | 15,16 | 15,16 | 15,17 | 15,17 | 16,18 | 16,18 | 16,18 | 14,14 | 14,14 | 17,17 | 17,17 | 15,16 | 15,16 |
